# Integrated Analysis of Behavioural and Health COVID-19 Data Combining Bayesian Networks and Structural Equation Models

**DOI:** 10.3390/ijerph19084859

**Published:** 2022-04-16

**Authors:** Ron S. Kenett, Giancarlo Manzi, Carmit Rapaport, Silvia Salini

**Affiliations:** 1 KPA Group and Samuel Neaman Institute, Raanana 43100, Israel; ron@kpa-group.com; 2 Data Science Research Centre, Department of Economics, Management and Quantitative Methods, University of Milan, 20122 Milan, Italy; giancarlo.manzi@unimi.it; 3 Department of Geography and Environmental Studies, University of Haifa, Haifa 3498838, Israel; carmit.rapaport@gmail.com; 4 NIRED—National Institute for Regulation of Emergency and Disaster, College of Law and Business, Ramat Gan 5110801, Israel

**Keywords:** Bayesian Networks, SEM, COVID-19 pandemic, integrated models

## Abstract

The response to the COVID-19 pandemic has been highly variable. Governments have applied different mitigation policies with varying effect on social and economic measures, over time. This article presents a methodology for examining the effect of mobility restriction measures and the association between health and population activity data. As case studies, we refer to the pre-vaccination experience in Italy and Israel. Facing the pandemic, Israel and Italy implemented different policy measures and experienced different population behavioral patterns. Data from these countries are used to demonstrate the proposed methodology. The analysis we introduce in this paper is a staged approach using Bayesian Networks and Structural Equations Models. The goal is to assess the impact of pandemic management and mitigation policies on pandemic spread and population activity. The proposed methodology models data from health registries and Google mobility data and then shows how decision makers can conduct scenario analyses to help design adequate pandemic management policies.

## 1. Introduction

The COVID-19 pandemic has far-reaching consequences for global, national and local economies. National non-pharmaceutical interventions (NPI) and policies to control the pandemic spread, such as lockdowns (local or general), movement restrictions, and massive testing to detect outbreaks, have been widely applied. However, given the human-to-human transmission of the SARS-CoV-2, the population’s behaviour, which includes compliance with official instructions as well as active protective measures, is a critical factor in pandemic management. The public’s adherence to the instructions, such as wearing face masks, social distancing and hygiene practices, requires a public behavioural change which cannot be taken for granted. Strictly enforcing such changes, on a national level, is almost impossible. Furthermore, disruptive pandemic management policies, such as national and local lockdowns, with frequent closures of air traffic, education institutions, and economic sectors, have severe economic and social consequences. These include increasing rates of domestic violence [1], unemployment [2], mental distress [3,4], and non-normative or addictive behaviours such as alcohol drinking and drugs use [5,6].

Governments’ policies of lockdowns and re-openings are determined according to a perceived “acceptable loss” that aims to minimize economic and social damage while saving lives [7]. Therefore, it has become clear that pandemic management must take in consideration social, public health, behavioural and economic factors, as well as monitoring of the population’s ability to maintain restrictions over time.

Given the critical role played by human behaviour in the pandemic, research studies have examined how policy and NPIs affect population morbidity, death rates and spreading of the disease. These include policies such as “stay at home” instructions, social distancing and closures of businesses and air traffic [8,9,10,11,12], as well as guidelines on taking protective actions such as using face masks [13]. Overall, research has indicated that NPIs’ effectiveness increases when applied in different ways. For example, Brauner et al. [14] found that prevention of social contacts at social places such as education institutions and restaurants, as well as small gatherings, were more effective in lowering transmission than lockdowns. Block et al. [15] show that reduced contact between people improves the effectiveness of social-distancing strategies. Changes over time in the effectiveness of the applied mobility restriction policies have also been studied [16].

In addition to official policies and regulations, population behaviour was found to be an important factor in disease transmission. Citizens voluntarily decreased their mobility, without official instructions to stay at home [17,18,19], and some groups even objected to the removal of restrictions [20]. Citizens’ compliance with stay-at-home policies [21] was associated with perceived risks [22]), trust in science and scientists [23], trust in the authorities [24], social capital [25] and political orientation [26]. Additionally, social responsibility in caring for relatives and community [27], future economic status [28] and messages of pro-social advantages of adhering to the instructions [29] were linked to higher levels of adherence to lockdown instructions. Taken together, these findings suggest that a range of factors, both at the individual and the social level, affect citizens’ compliance with protection instructions. This emphasizes the importance of integrating behavioural and policy measures in pandemic management. The public plays a critical role in pandemic management, as both a “client” who receives and consumes health services from the government, and therefore is susceptible to its instructions and regulations, and as the “pandemic management target” to the behaviour of which all pandemic management steps are subordinated, capacities, compliance and resilience. The current study examines these mutual interactions, to assess their effects and consequences on both the public and the heath indicators.

In this paper, we present a methodology for the analysis of data on population behaviour and health, such as the number of deaths and patients in intensive care units (ICU) in the COVID-19 pandemic. Specifically, we consider the impact of lockdowns and mobility restrictions, as reflected by public behaviour mobility data. The challenge we address here is combining these sources of data into a unified system that provides support and information to decision makers. Our goal is to identify links between population behaviour (activity), COVID-19 health data and policy decisions to find what are the effects, over time, and when applying various NPIs. The data analysis requires calibration since a simple temporal consideration is not adequate. Additionally, our analysis shows the mutual effects of human behaviour and the health data to explore the complexity of pandemic management and discover the most effective ways to reduce hospitalisation rates and death rates.

We consider here a multivariate exploratory approach in an “ensemble” fashion, combining outcomes from Bayesian Networks (BN) and structural equation models (SEM). We first apply our ensemble approach to pre-vaccination pandemic data from Italy. Then, as a confirmatory step, we apply it to pandemic data from Israel. This generalizes the methodology to a context with a different pandemic history and health infrastructure and in terms of timing and health policy. This allows us to validate our approach by considering changes in data structure and application context. We compare the results from such an integrated analysis in Israel and Italy, pointing out the differences found in these two countries. The combination of BN and SEM tools highlights the different policies adopted by the two countries and suggests that these tools are sufficiently flexible to be applied in different contexts. Finally, in order to provide the capability to evaluate alternative scenarios, we update the BNs, following discretisation of the original data using country-specific thresholds applied both in Italy and Israel. The thresholds on health indicators were set to safeguard hospitalisation capacity in the two countries.

## 2. Pandemic Management Policies in Italy

The first COVID-19 cases reported in Italy—two Chinese tourists travelling across Italy—were discovered on 9 January 2020. These cases did not trigger any outbreak as the infected persons were immediately isolated and hospitalised. The first real outbreak was reported in the village of Codogno in Northern Italy at the end of February 2020. This was the first serious hot-spot of the COVID-19 pandemic outside China which spread in Europe and other Western countries, including the US. On 4 March 2020 all schools and universities were closed in Italy, and on 11 March 2020, a national lockdown was introduced. On 3 June 2020, the national lockdown was eased and free movement between regions was allowed and schools and shops reopened.

Following the summer, when reopening of night clubs, gyms and free movements for touristic reasons was allowed, in September 2020, a second wave of the pandemic started, and new restrictions were introduced in October 2020. On 6 November 2020, a regional “traffic light system” was introduced in Italy according to the current pandemic severity level: (i) “yellow” areas where all shopping malls and supermarkets are closed during weekends. Culture and recreation places (museums and cinemas) are closed, and restaurants are open until 6 p.m. Only elementary and primary schools are open, while universities are open with 50% of the students allowed to attend classes, a curfew from 10 p.m. to 6 a.m. is also set; (ii) “orange” areas where all restaurants are closed all day, public transport is open on a 50% filling capacity level, universities are closed, only elementary and primary schools are open, malls are closed during the weekends, gyms, cinemas theatres and museums are closed; curfew is still in place and movements are allowed only locally; (iii) “red” areas where no movements are allowed apart from emergency and special cases, all shops, school, universities, restaurants, cinemas, theatres, museums, gyms are closed, with the exception of essential services; curfew from 10 p.m. to 6 a.m. is still in place. In mid-December 2020, the third wave of pandemic started, leading in March 2021 to a situation where almost all Italian regions were under “red area” alert.

On 27 December 2020, the first inoculation was administered in Italy. The government’s vaccination plan was to administer the vaccine first to healthcare personnel, then to the elderly, then to school and university staff, and ultimately to the rest of the population. However, as the Italian health system is region-based, the vaccination plan was affected by a lack of coordination between the central government and the regional administrations. In addition, the shortage of vaccine doses generated stress in respecting the clauses of the contracts with the EU regarding the planned deliveries. The vaccination plan was lagging behind the initial scheduled timeline and, in March 2021, only 9% of the Italian population received the first vaccine dose.

## 3. Methods

### 3.1. Analysis Strategy

**Figure 1:**
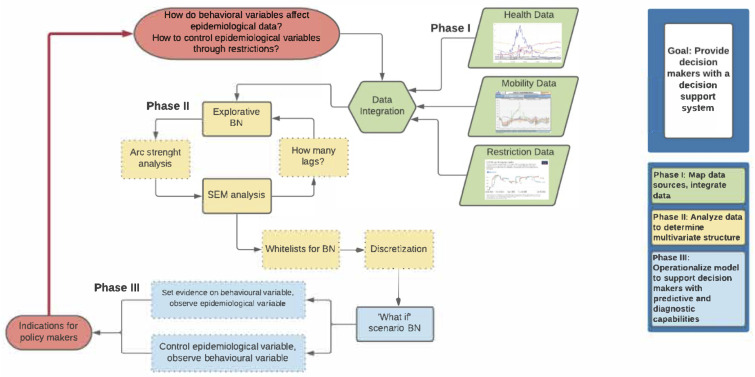
Flow chart describing the ensemble analysis.

We modelled the multivariate structure in the daily health indicators and the population activity data, referring to major policy decisions (local/national lockdown, reopening). The analysis is focused on Italy which was first in experiencing the pandemic as a national threat to its healthcare system. The period we are considering precedes the start of the vaccination program in Italy. The statistical analysis we conducted includes modelling based on methods designed to enhance information quality, including Bayesian Networks (BN) and Structural Equation Models (SEM). Each modelling approach provides complementary features. The idea is to conduct an ensemble type analysis combining outcomes from various models [30]. The analysis flows through three sequential steps starting from an initial descriptive BN analysis, aiming at finding the most important relationships with the highest arc strengths between variables. This, in turn, is the basis for a SEM analysis where relationships with the most important latent variables are found. The third stage is a “what-if scenario” evaluation using BN analysis where assumptions of multiple configurations are considered. This is described in Figure 1. In the final phase, when various scenarios are investigated, the data are discretised using local thresholds in order to enhance the intelligibility of the scenarios.

### 3.2. Italian Case Study

The method applied in this work combines official COVID-19 daily health data from the Italian Ministry of Health, starting from 24 February 2020 until 21 January 2021. The data was downloaded on 12 November 2021. All the graphs, tables and links that are presented in this work refer to that date. Variables included in the study are: number of COVID-19 hospitalised patients, number of severe condition patients (in ICUs), and number of COVID-19-attributed deaths per day. We also use Google Mobility data (https://www.google.com/covid19/mobility/ (accessed on 26 February 2022)) for Italy for the same dates. Variables included mobility indicators for retail and recreation, grocery and pharmacy, parks, transit stations, workplaces, and residential buildings. Mobility variables show the percent change from pre-pandemic baseline. We also include restriction variables from the University of Oxford’s COVID-19 government response tracker research group (https://www.bsg.ox.ac.uk/research/research-projects/covid-19-government-response-tracker (accessed on 26 February 2022)).

We use BN analysis to map the relationships between epidemiological and behavioural variables. Death statistics are used to assess the impact of the pandemic and the number of infected people, as reported on a daily basis. Our analysis proceeds through the following steps:

- First, we performed an initial exploratory BN for both Israel and Italy in order to describe the relationships among variables. In this step, we also performed a between-node strength analysis to derive a hierarchy of the most important variables affecting ICU, deaths and hospitalisation;
- Then, we identified from the previous BN the most significant nodes and used these nodes in a SEM to study the significance of the relations among nodes;
- Finally, we built up a “what-if scenario” BN analysis to model the most epidemiological variables, i.e., ICU, death and hospitalisation, in order to analyse the dynamics of the pandemic with respect to behavioural variables in the two countries.

To conduct the analyses, we used the package R (www.R-project.org/ (accessed on 26 February 2022)), and its libraries *bnlearn* and *bnviewer* for BNs, and JMP Pro 16 (www.jmp.com (accessed on 26 February 2022)) for SEM. We provide below a brief introduction to BN and SEM pointing to references for more details.

### 3.3. Bayesian Networks (BNs)

Formally, BNs are direct acyclic graphs (DAG) whose nodes represent random variables in the Bayesian sense: they can be observable quantities, latent variables, unknown parameters or hypotheses [31]. The arcs represent conditions of dependence; the nodes that are not connected represent variables that are conditionally independent of each other. Each node is associated with a probability function which takes as input a particular set of values for the variables of the parent node and returns the probability of the variable represented by the node. There are efficient algorithms that perform inference and learning starting from BNs. A BN enables the effective representation and computation of a joint probability distribution (JPD) over a set of random variables. The DAG’s structure is defined by a set of nodes, representing random variables and plotted by labelled circles, as well as a set of arcs representing direct dependencies among the variables and plotted by arrows. Although the arrows represent direct causal connections between the variables, under some conditions, the reasoning process can operate on a BN by propagating information in any direction. A BN reflects a simple conditional independence statement, namely, that each variable, given the state of its parents, is independent of its non-descendants in the graph. This property is used to reduce, sometimes significantly, the number of parameters that are required to characterize the JPD. This reduction provides an efficient method to compute the posterior probabilities given the evidence in the data [32]. It is even possible to extract more conditional independencies from the DAG’s structure using the *d-separation* procedure. Suppose, for example, that we have to check whether P(A|BCD)=P(A|CD), i.e., to see if A and B are independent given C and D. Then, the following d-separation procedure can be applied:

1. Obtain the ancestral graph, i.e., a reduced version of the graph pertaining to all the the variables in the above probability expression;
2. “Moralized” the graph obtained in 1. by “marring” the parents. This will result in an undirected edge between each pair of variables that have a common child;
3. “Disorient” the graph by using undirected edges instead of directed edges;
4. If the independence question had any “given variables”, erase those variables from the graph and all their connections;
5. If the variables become disconnected, they are independent. Alternatively, if the variables result are connected, they are not guaranteed to be independent. Finally, if one or both of the variables are missing, they are independent.

In this way, independence relations are determined in a more insightful fashion. For example, it can be easily established if two variables having no common ancestors in the graph and therefore appearing marginally independent can become dependent given their common child node [33].

### 3.4. Structural Equation Models (SEM)

SEM is a set of statistical techniques used to measure and analyse the relationships of observed and latent variables [34]. It examines linear causal relationships among variables, while simultaneously accounting for measurement error. SEM is widely used in the social sciences and psychology. It provides a flexible framework for developing and analysing complex relationships among multiple variables that allow researchers to test the validity of a theory using empirical models. A discussion about SEM and its connection with causal models is reported in [35].

Structural equation modelling involves the specification of an underpinning linear regression model incorporating the structural relationships between unobserved and latent variables, together with a number of observed or measured indicator variables. Structural equation models assume a structure among a set of latent variables and observed variables. The latent variables appear as linear combinations of observed variables. As latent variables are, by definition, unobserved, their measurement must be obtained indirectly. This is achieved by linking one or more observed variables to each unobserved variable. A fully specified structural equation model involves presenting an interplay between a large number of observed and unobserved variables, and residual and error terms.

### 3.5. Combining BN and SEM

Gupta and Kim [36] propose linking BN to SEM, which presents an advantage in testing causal relationships between factors. The capability of SEM in empirical validation, combined with the prediction and diagnosis capabilities of Bayesian modelling, facilitates effective decision making from identification of causal relationships to decision support. An interesting application that combines SEM and BN is in [37].

In this study, as illustrated in the flow chart of Figure 1, we use SEM and BN in a combined way. First, we used BN to define how late to consider the health variables through the strength analysis. Then, we estimate a SEM model that considers the previously identified variables. On the basis of the significance emerged in the SEM model we establish the variables to be included in the discrete networks and the whitelists to make the what-if scenarios.

### 3.6. Study Variables

The integrated analysis considers three groups of variables collected daily in Italy from 24 February 2020 to 1 January 2021. These are: (i) mobility variables from Google Community mobility reports; (ii) Pandemic management NPI variables from the University of Oxford’s COVID-19 government response tracker; and (iii) health variables from the Italian Ministry of Health.

1. Mobility variables.
  Mobility variables are reported as changes in mobility with respect to a pre-COVID-19 baseline. We include mobility changes in retail and recreation, grocery and pharmacy, parks, transit stations, workplaces, and residential buildings. Restriction variables are ordinal variables (from 0 = “no measures at all” to a maximum of 3 = “required closing of all events”) expressing the intensity of governmental measures to tackle the spread of the virus. These were related to school closing, workplace closing, gathering restrictions, transport closing, stay-at-home restrictions, international movement restrictions, internal movement restrictions. We also consider a stringency index variable ranging from 0 to 100, as a weighted average of the restriction variables.
2. Health variables.
  These include the number of daily COVID-19-related deaths, the number of daily hospitalisations due to COVID-19 symptoms and the number of daily COVID-19 ICU admissions [38]. We also consider a variable labelled “wave” to detect different behaviour observed in subperiods of different intensity levels in each country within the period of observation.
3. Pandemic management NPI variables.
  We consider the following subperiods: (i) Italy’s *subperiod 1*, from 24 February 2020 to 15 June 2020, corresponding to the “Italian first pandemic wave” lasting from the very first case detected in Italy to the end of the first lockdown; (ii) Italy’s *subperiod 2*, from 16 June 2020 to 15 September 2020, corresponding to the first period of pandemic ease, lasting from the end of the first lockdown to the initial new curbs introduced for the “Italian second pandemic wave”; (iii) Italy’s *subperiod 3*, from 16 September 2020 to 8 October 2020, corresponding to the beginning of the “Italian second pandemic wave”, lasting from the introduction of new measures until the introduction of the so-called “colour system”, i.e., a four-level system for regional restrictions from “white” = “no restrictions” to “red” = “full lockdown”; (iv) Italy’s *subperiod 4*, from 9 October 2020 to 11 November 2020, corresponding to the most acute pre-vaccination period of pandemic in Italy; (v) Italy’s *subperiod 5*, from 12 November 2021 to 21 January 2021, from the ease of the second pandemic wave to the initial vaccination campaign in Italy, which started in late December 2020 but had an effect on protecting the population at the end of January 2021.

## 4. Results

### 4.1. Bayesian Network Analysis

The main group of variables of interest is one of health variables. First, in the BN, we blacklist the arcs between variables within each group. Then, we create structures using Hill-climbing BNs, focusing on variables (nodes) with a link to the health variables.

Maximum Likelihood parameter estimation is used for mixed-type data and Bayesian parameter estimation is used for discrete data.

Hill-climbing algorithms are particularly popular because of their good trade-off between computational demands and the quality of the models learned. It is important to verify whether the chosen structure and, therefore, its edges, are robust and do not depend on the chosen algorithm. The choice of a robust BN is a complex problem without an easily derivable analytical solution. In [39], different algorithms are presented and discussed and no method emerges absolutely better than another. For complex data, it can be shown that score-based algorithms produce large networks, in which higher-order dependencies are profoundly represented. This is an interesting aspect for our scopes. In order not to risk obtaining a network with a non-robust structure, following [40], we have in any case applied all the algorithms present in the *bnlearn* R library. Here https://unimibox.unimi.it/index.php/s/LLeGPsmeSdxeimb (accessed on 26 February 2022), it is possible to see that the selected arcs are present in at least four algorithms out of a dozen present in the library.

**Figure 2:**
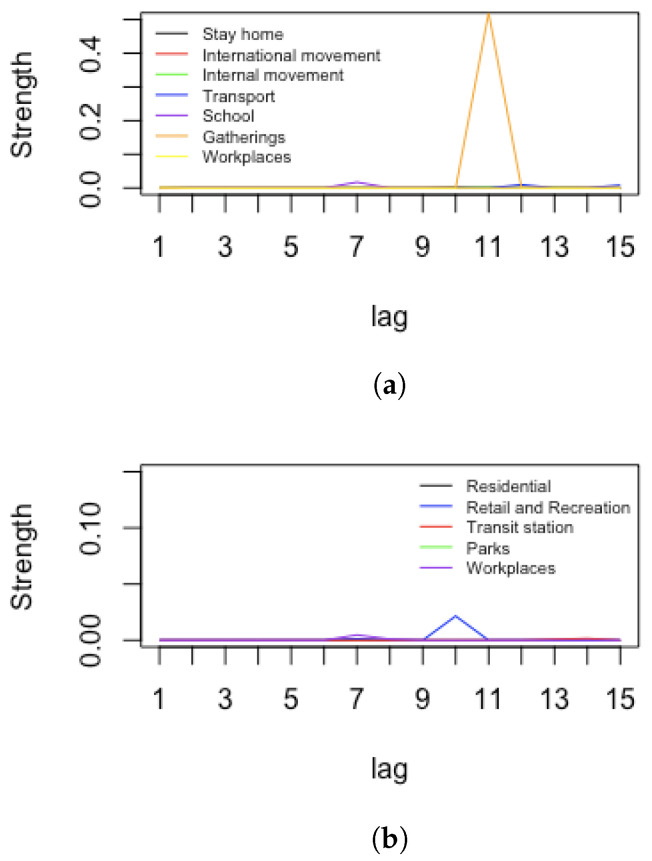
Strength analysis for Italy. Arc strengths according to different lags. Restriction nodes vs. hospitalisation node (**a**). Mobility nodes vs. hospitalisations node (**b**).

Since the effect of mobility and restrictions are observed with delay, after investigating several possible lags, we decided to use 10-day lags for the hospitalisations, 15 days for the number of people in ICUs and 20 days for the number of deaths with respect to the time where measures were implemented. This followed an arc strength analysis of arcs pointing towards the health nodes, looking for the highest average strength among different lags. Figure 2 presents arc strength levels between (respectively) restriction and mobility nodes vs. the hospitalisation node, according to different lags. The same plots have been obtained for ICU numbers and deaths, where the more reasonable lags were 15 days and 20 days, respectively.

**Figure 3:**
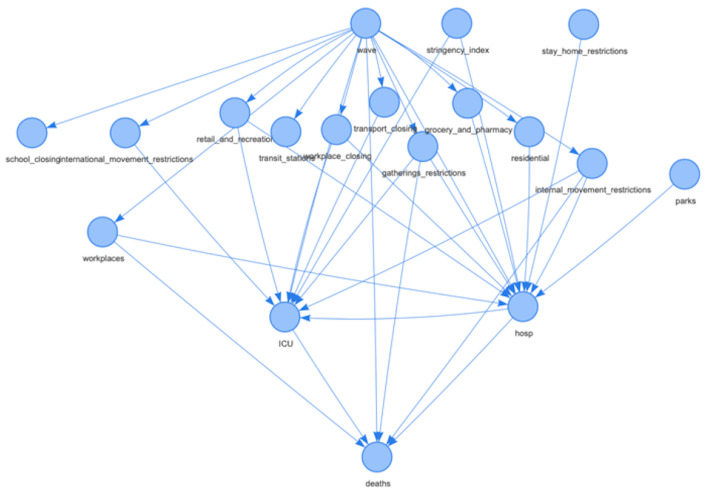
BN structure for Italy. The figure is downloadable at https://tinyurl.com/rbexhtww (accessed on 26 February 2022).

**Figure 4:**
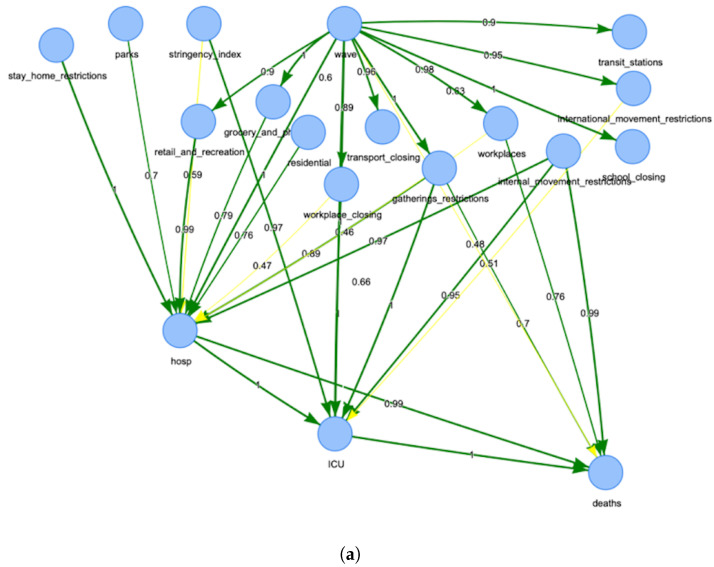

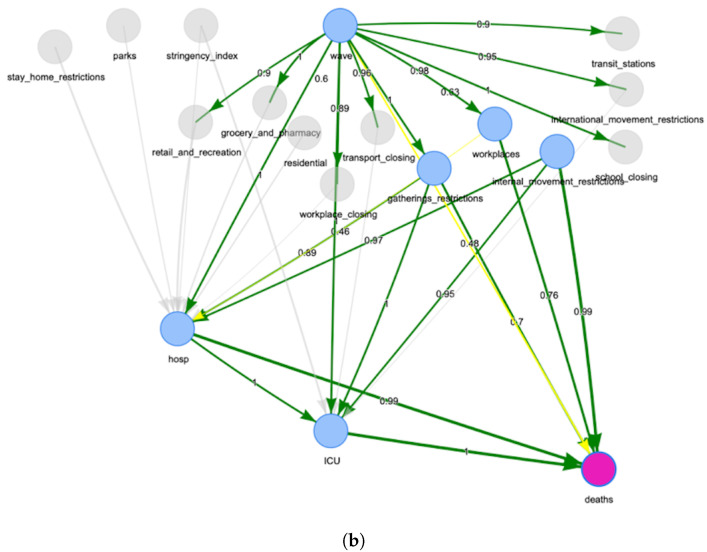
Network for Italy obtained using the bootstrap. (**a**) Overall network for Italy; (**b**) connections to the node “*deaths*” in the network. The figures are downloadable at https://tinyurl.com/rbexhtww (accessed on 26 February 2022).

Figure 3 reports the derived BNs for Italy. Measuring the degree of confidence in a graphical structure of a BN is a key problem in inference. Figure 4 reports the same network, but with the arc strength analysis obtained by applying non-parametric bootstrap to the data and estimating the relative frequency of the feature of interest [41]. In particular, Figure 4b displays the node‘ *deaths*’ highlighted together with its parent nodes and the relative arcs with the estimated strength for the network. All graphs in Figure 3 and Figure 4 can be downloaded in *html* format from the repository at the link https://tinyurl.com/rbexhtww (accessed on 26 February 2022) where it is also possible to browse and explore them interactively.

In addition to the ICU node, the node “deaths” has three strong direct arcs pointing to it. Hospitalisation can lead strongly and directly to death in Italy (0.99 strength). Among nodes representing restrictions gathering restrictions, workplace restrictions and internal movement restrictions have a huge direct effect on deaths. Moreover, there is a direct link from hospitalisation to the death node. This could be explained by the low structural level of the healthcare system in Italy and the lack of ICU beds. Moreover, the node “wave” seems to be quite important. This could mean that in Italy there has been a huge change in citizens’ behaviour over time, in terms of complying with the instructions across the waves.

We will explore again the BN originated after a SEM analysis and a discretization of the variables to see whether or not these initial considerations will be confirmed through a “what-if” analysis.

### 4.2. Structural Equation Modelling

After identifying the arcs between nodes with the BN, we use SEM to understand which arcs were significant and also to check the robustness of the bootstrap approach to BNs. In the discrete networks that we used for the scenarios, we set white-lists based on the significant arcs in the SEM model. White-lists are arcs manually imposed on the BN.

**Figure 5:**
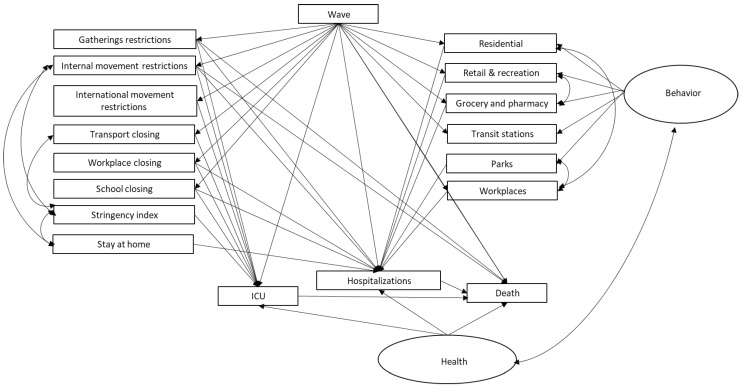
Path diagram for the Italian data.

In applying SEM, we introduce two latent variables: *behave*, representing the behavioural data and *health*, driving the health-related data (number of death cases with a lag of 20 days, number of hospitalized in ICU with lag 15 days, and number of hospitalized patients (with a lag of 10 days). Figure 5 presents the SEM path diagram for Italy. Table 1 presents the loadings, regressions and covariances for the Italian data. Table 2 presents the summary of model fit indices. We find an acceptable goodness of fit of the model and a CFI of 0.77. The results show that the population behaviour was found to be significantly predicted by wave. However, over time, the effect of the “wave” is reduced, as attendance at public places, such as workplaces, transit stations, groceries and pharmacies, and retail and recreation, is not reduced. Moreover, hospitalisation decreased death cases. Restrictions, such as internal lockdowns, international border closings and gathering restrictions led to an increase in health measures. The latent variables, *health* and *behave* were significantly and negatively correlated—when health measures increase, the public reacts by decreasing behavioural activities and vice versa.

### 4.3. “What-If” Scenario BN Analysis and Strength Analysis

We now present a “what-if” scenario-based BN analysis where we discretise the health variables and obtain results by setting the categories of some mobility or restriction variables to specific values which are of interest. Moreover, inverse inference can be achieved by fixing the levels of the health variables and seeing what happens to the parent nodes. Discrete network white-lists were set up based on the SEM results reported in Table 1. This conditioning provides decision makers with a powerful decision support tool used in policy-making discussions.

**Figure 6:**
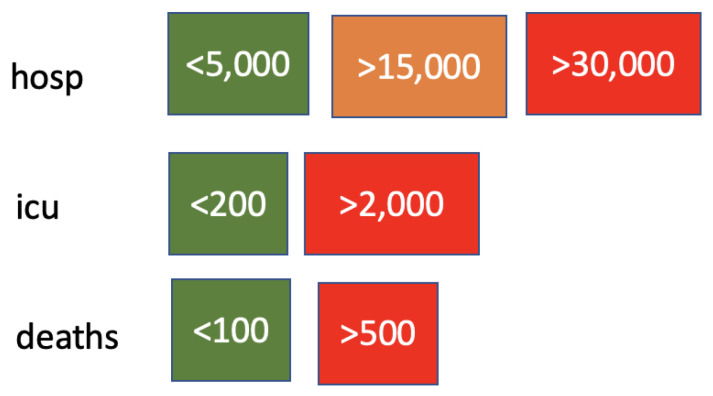
Cut-off points for health variables.

**Figure 7:**
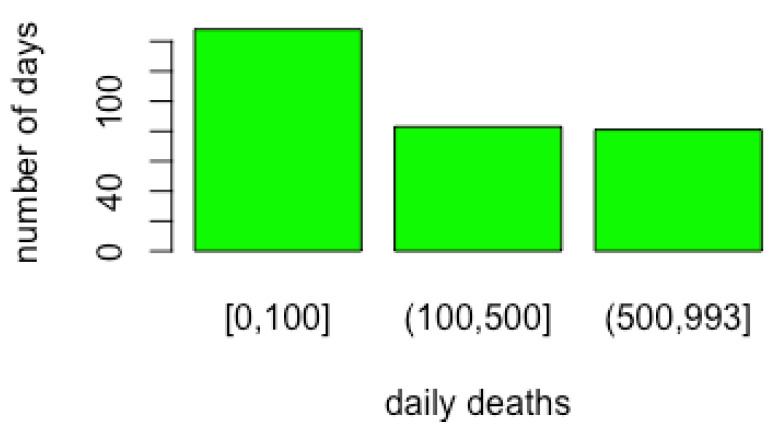
Discretisation results for COVID-19 death variables in Italy. Class limits are reported on the *x*-axis.

Health variables are discretised according to the cut-offs reported in Figure 6. Cut-off threshold values were chosen to represent the healthcare system and the size of the country. Figure 7 shows the distribution of daily deaths in Italy in terms of number of days distributions in the three classes formed by the cut-off points. The restriction variables are ordinal, so they are not discretised. For the behaviour and activity variables, Hartemink’s algorithm [42] is used with a number of cut-off values equal to 3.

**Figure 8:**
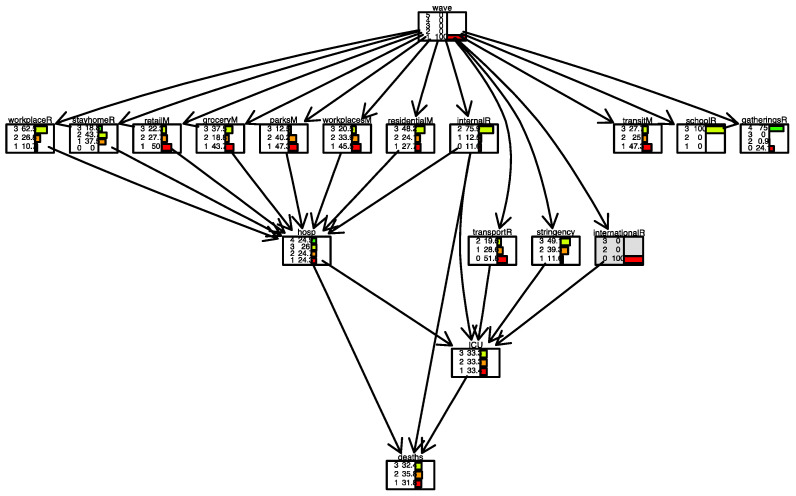
Scenario for Italy when *international movements restriction* is set to 0. International movement restriction node is highlighted in grey.

**Figure 9:**
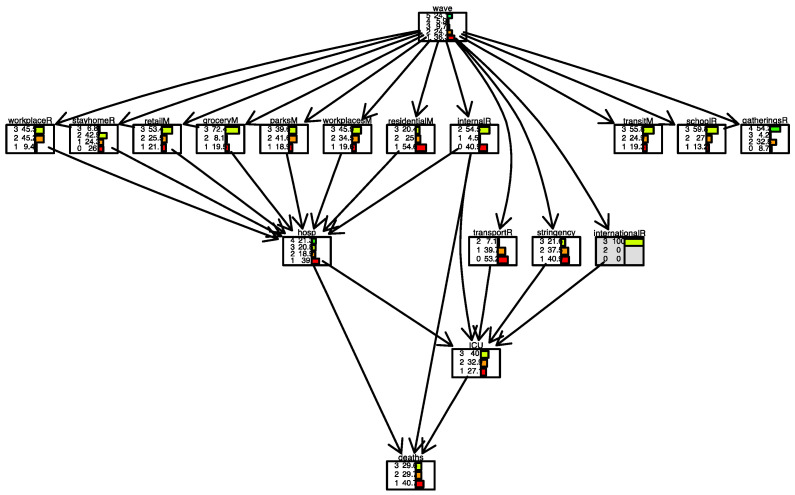
Scenario for Italy when *international movements restriction* is set to 3. International movement restriction node is highlighted in grey.

Figure 8 and Figure 9 show two scenarios for Italy. In the first scenario, we set evidence as the lowest level of the *international movement restriction* variable. In the second scenario, we set evidence as the highest level of the *international movement restriction* variable.

The distribution of the variable *deaths* changes in the following conditions. When restrictions are present, the number of days when the number of daily deaths is low (less than 100 cases) is 40%. It is 32% when restrictions are not present.

**Figure 10:**
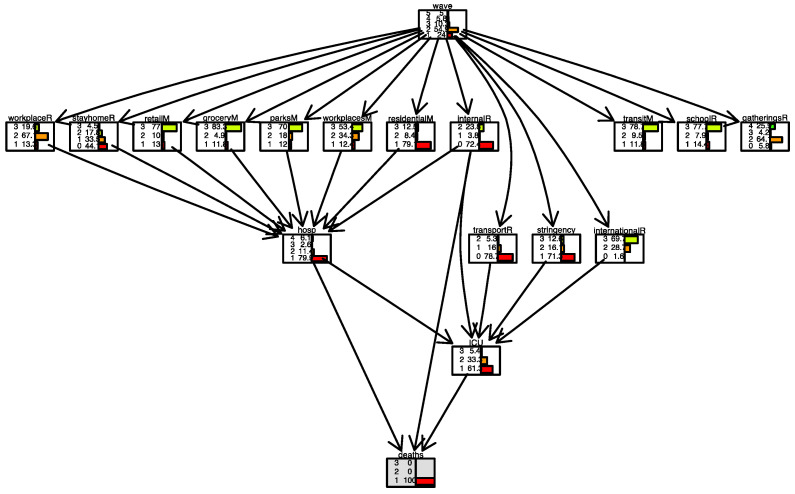
Scenario for Italy when daily deaths are less than 100 (level 1). Deaths node is highlighted in grey.

**Figure 11:**
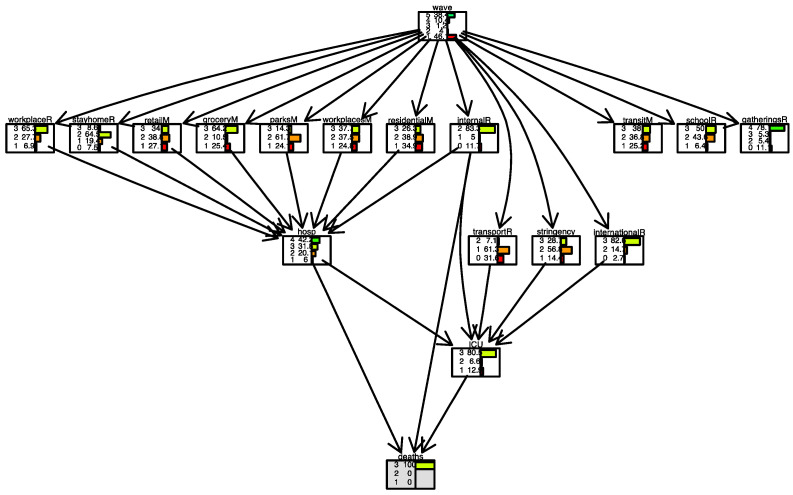
Scenario for Italy when daily deaths are larger than 500 (level 3). The deaths node is highlighted in grey.

In Figure 10 and Figure 11, conditional inference is applied to the case of Italy. With daily deaths level remained fixed, we observe what happens in the parent nodes. In Figure 10, the scenario is one where the number of daily deaths is always less than 100. In Figure 11, the scenario is one where the number of daily deaths is always larger than 500. In this latter case, we observe, for example, that the lowest level in the *stay-home restriction* is present only in 7.5% of the days. In contrast, when daily deaths are always lower than 100 (Figure 10), the lowest level in the *stay-home restriction* is present on 44.1% of the days. We also note that *residential mobility* was high (level 3) in 26.3% of the days when daily deaths were more than 500 and only 12.5% of days when daily deaths were less than 100.

Some restriction measure variables behave in unexpected directions. For example, *mobility behaviour* changes with no direct reference to deaths, and the *internal movement restriction* variable changed from 23.8% at the maximum rate (=2) when deaths were minimum (=1) to 83.3% when deaths were maximum (=3), meaning that fewer deaths imply less movement. This could be explained by the fact that in Italy restriction measures have been in places for long periods, even when deaths increased or decreased. Therefore, movements remained low even with a low number of deaths or high with a large number of deaths.

## 5. Validating the Ensemble Approach

In this section, we validate the proposed ensemble method by applying it to another country, Israel. This provides a context similar to Italy with respect to the pandemic from the point of view of policy decisions, but different in terms of hospitalisations, ICU occupancy and COVID-19 deaths.

**Figure 12:**
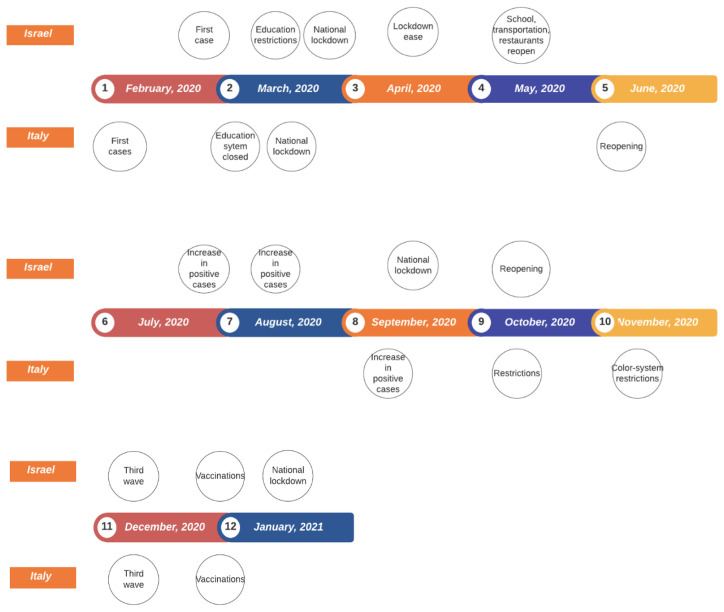
Main pandemic events in Italy and Israel.

The first case of COVID-19 in Israel was discovered on 28 February 2020. The Jewish holiday Purim was observed on 10–11 March 2020, and it involves mass gatherings and public celebrations. This caused a sharp increase in the number of the positive cases, which led to drastic movement restrictions in the entire country. On 14 March 2020, the academic and school systems were closed and gatherings of more than 10 persons were forbidden. On 25 March 2020, Israel underwent a general lockdown. In 9 April 2020, due to a decrease in the number of ICU patients and those hospitalised in severe condition, a first easing of the lockdown allowed for sport activities to be practised more than 500 m from one’s home. In May 2020, schools, public transportation and public places such as restaurants reopened. In July and August, health measures indicated a new outbreak, and new restrictions were placed until a second general lockdown, which started on 18 September 2020. On 17 October 2020, public spaces and schools started to reopen against the recommendations of health professionals. A rising number of daily positive cases, number of deaths and severe patients led to a third wave (starting at the beginning of December 2020). On 8 January 2021, Israel was placed under a third national lockdown that lasted one month. From 20 December 2020, a massive vaccination operation was conducted with world-record-breaking records numbers of vaccinated people. The data considered here reflect the pre-vaccination period. Figure 12 shows the timing and the main pandemic events in both Italy and Israel over time.

For Israel, we consider the following sub-periods: *Israel’s sub-period 1*, from 24 February 2020 to 17 March 2020, since the first case was detected until the first lockdown, *Israel’s sub-period 2*, from 18 March 2020 to 26 April 2020, corresponding to the so-called first wave of the pandemic in Israel, in which restrictions were imposed to the population, *Israel’s sub-period 3*, from 9 June 2020 to 3 November 2020, corresponding to the second pandemic wave in Israel, *Israel’s sub-period 4*, from 4 November 2020 to 21 January 2021, corresponding to the third pandemic wave in Israel and during which a massive vaccine campaign started.

**Figure 13:**
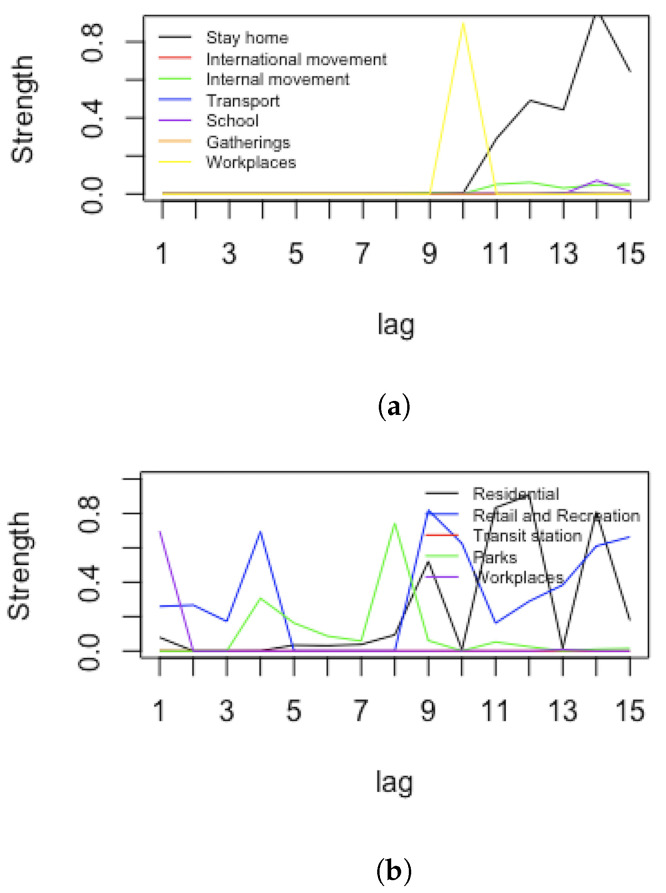
Strength analysis for Israel. Arc strengths according to different lags. Restriction nodes vs. hospitalisations node (**a**) and mobility nodes vs. hospitalizations node (**b**).

Figure 13 reports an arc strength analysis for Israel. Apart from the *stay-at-home* restriction, all other restrictions were in line with those in Italy with respect to hospitalization. For mobility nodes versus hospitalization, the highest strength peaks are reached after a lag of 9 days. Similar results are obtained for restrictions and mobility versus ICU patients and for restrictions and mobility versus deaths. Therefore, for Israel, we also take a 10-day lag for the hospitalisations, 15-day lag for the number ICUs and a 20-day lag for the number of deaths with respect to the time when measures were implemented.

**Figure 14:**
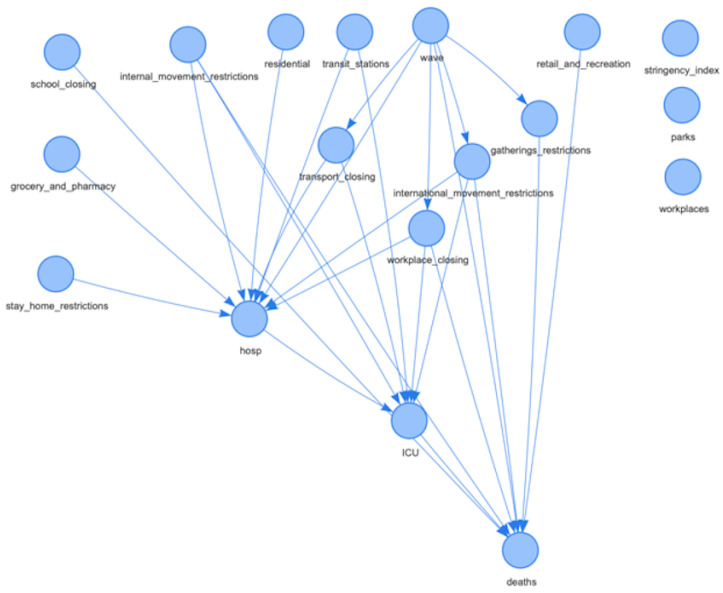
BN structure for Israel. The figure is downloadable from https://tinyurl.com/rbexhtww (accessed on 26 February 2022).

**Figure 15:**
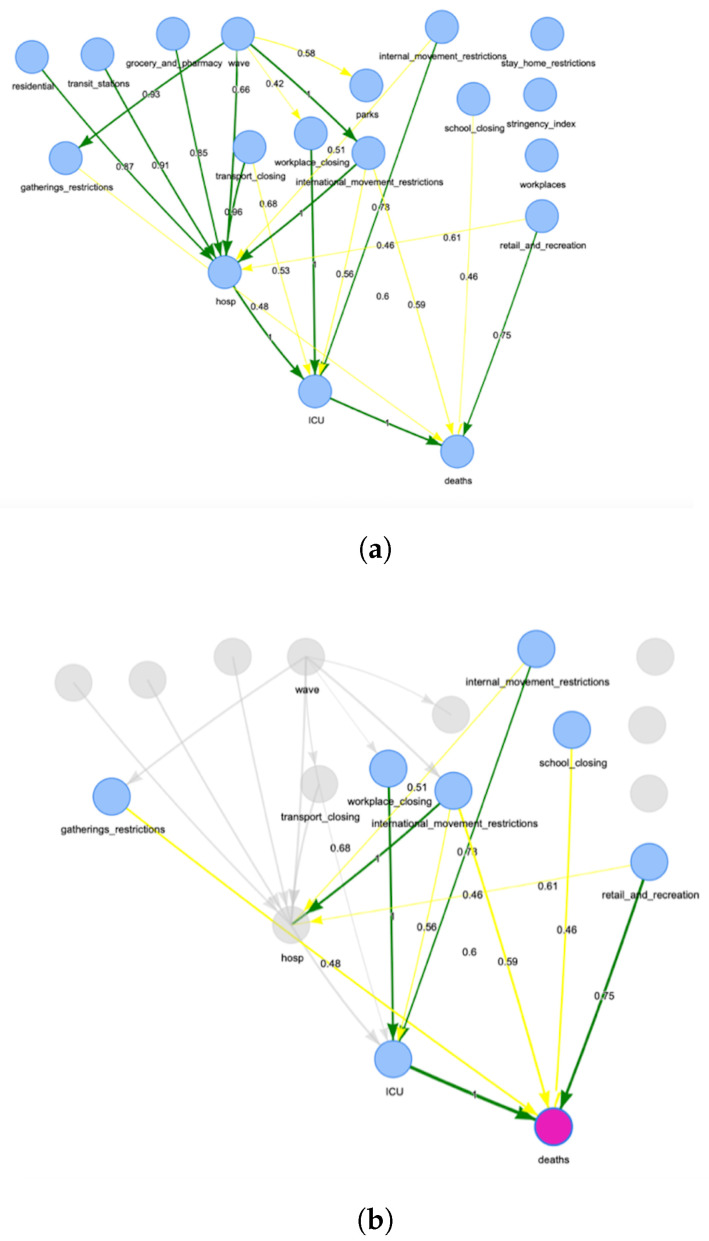
Network for Israel obtained using the bootstrap. (**a**) overall network; (**b**) connections to the node ‘*deaths*’ in the network. The figures are downloadable at https://tinyurl.com/rbexhtww (accessed on 26 February 2022).

Figure 14 reports the derived BN for Israel. Measuring the degree of confidence in a graphical structure of a BN is a key problem in inference. Figure 15 reports the same network, but with the arc strength analysis obtained by applying non-parametric bootstrap to the data and estimating the relative frequency of the feature of interest, as applied to the Italian case. Moreover, Figure 15a,b display the node ’deaths’ highlighted together with its parent nodes and the relative arcs with the estimated strength for the networks for Italy and Israel, respectively.

**Figure 16:**
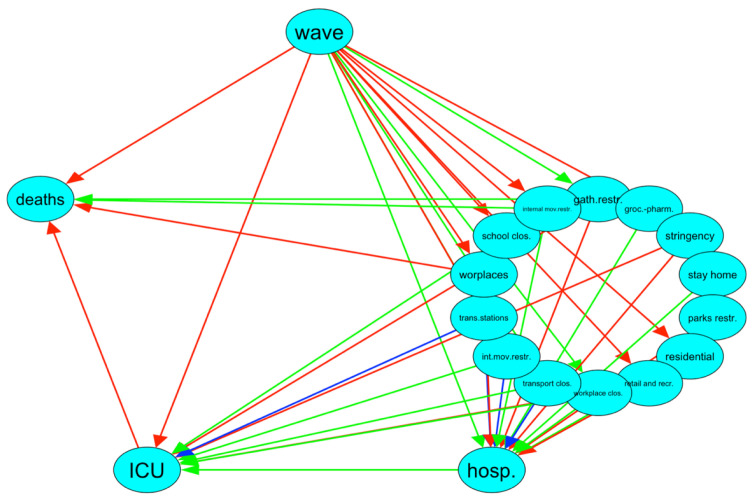
Comparison of lagged BNs for Italy and Israel, Italy is the target BN, Israel is the current BN. Green represents true positive, blue represents false positive and dashed red represents false negative. The resulting Hamming index is 23.

To confirm what we found in the exploratory BNs for both countries, in Figure 16, we compare the BNs of Italy and Israel. Italy is the target BN, Israel is the current BN. Green arcs are true-positive arcs, i.e., arcs which are present in both BNs, blue arcs are false-positive arcs, i.e., arcs present in current BN, but not present in the target BN, and red arcs are false-negative arcs, i.e., arcs not present in current BN but present in the target BN. There are six true-positive arcs leading to the hospitalization node, from the international movement closing node, internal movement closing node, workplaces closing node, residential mobility node, stay home restriction node, grocery and pharmacy mobility node. As for the ICU node, there is one true-positive arc from hospitalization (as expected), one from the transport closing node, one from the internal movement restriction node, one from the workplace closing node and one from the international movement restriction node. Finally, there is only one direct true-positive arc from the hospitalisation to the death node (as expected), and from the gatherings restriction node and one from the internal movement restriction node. The Hamming distance is calculated, considering each arc as a string. The classical Hamming distance between two equal-length strings of symbols is the number of positions at which the corresponding symbols are different. The Hamming index, i.e., the number of false positive and false negative arcs in this comparison, is equal to 23.

**Figure 17:**
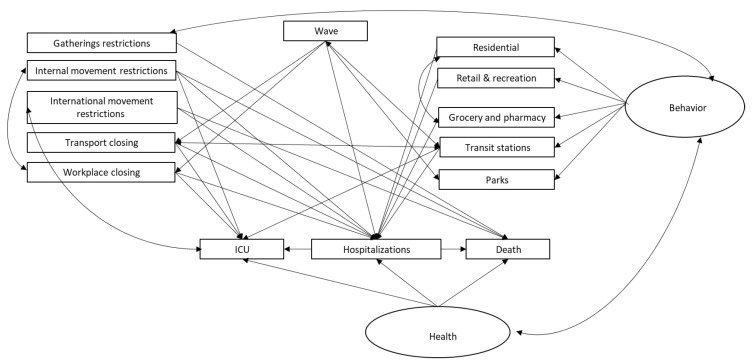
Path diagram for the Israeli data.

Figure 17 presents the SEM path diagram for Israel, and Table 3 presents the loadings, regressions and the covariances for the Israeli data. Data from Israel show a direct effect of restrictions on the health outcomes. International movement restrictions reduced the number of hospitalised and death cases. However, it was also found that transport closing decreased ICU cases, but increased hospitalisations. In terms of population behaviour, visits in transit stations decreased hospitalisations, while visits in grocery stores and pharmacies increased it. Table 4 presents measures of fit for the Israeli data. The SEM results confirm the significant arcs of the BN. The SEM comparative fit indices (CFI) are relatively low, but they need to be considered in the context of the overall analysis workflow and not as a stand-alone measure.

**Figure 18:**
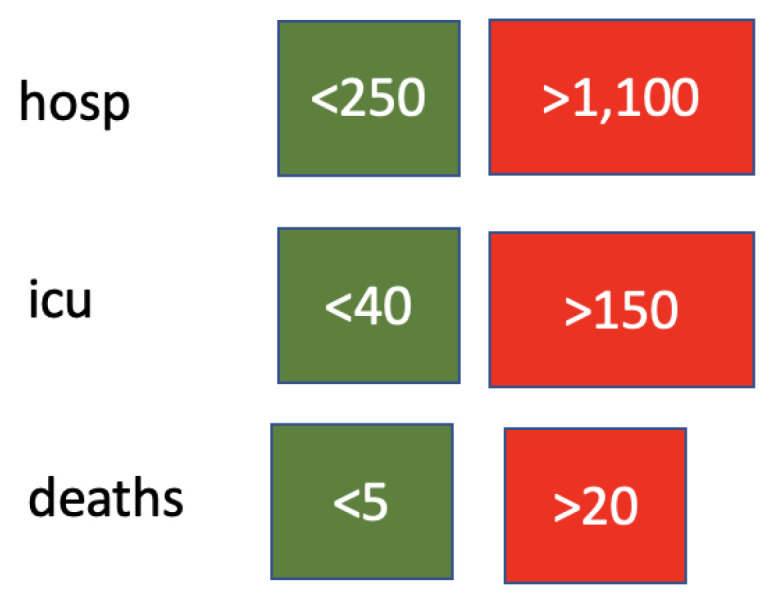
Cut-off points for health variables for Israel.

**Figure 19:**
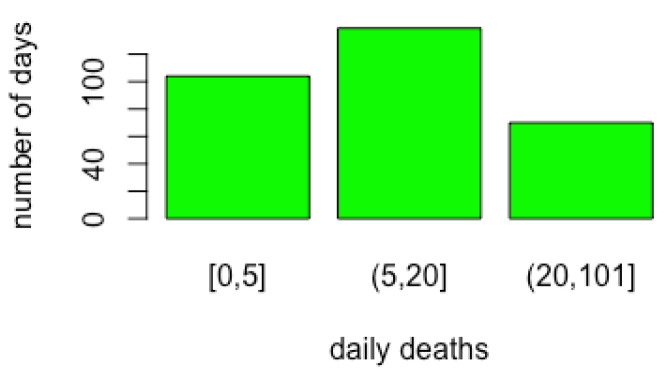
Discretization results for COVID-19 death variables in in Israel. Class limits are reported on the *x*-axis.

Health variables were discretised according to the cut-offs reported in Figure 18. Cut-off threshold values were chosen to represent the healthcare system and the population size in Israel. Figure 19 shows the distribution of daily deaths in Israel in terms of number of day distributions in the three classes formed by the cut-off points. Restriction variables are ordinal, so they are again not discretised. As for Italy, for the behaviour/activity variables, the Hartemink algorithm [42] is used with a number of cut-offs equal to 3. As a result, cut-off levels are lower in Israel than in Italy because of the different population size.

**Figure 20:**
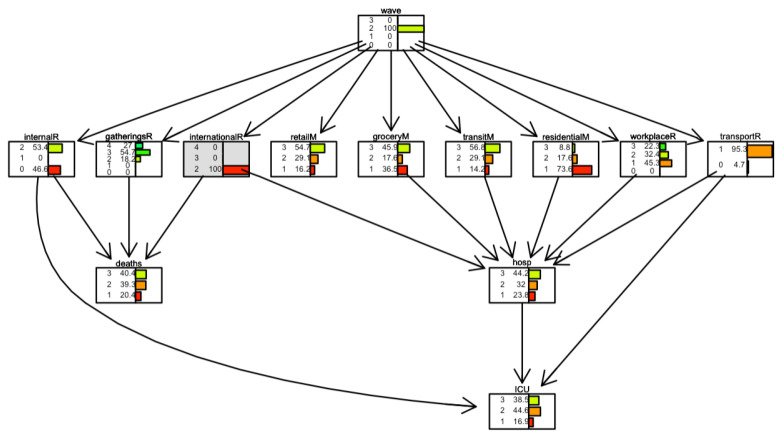
Scenario for Israel when *international movements restriction* is set to 2. The international movement restriction node is highlighted in grey.

**Figure 21:**
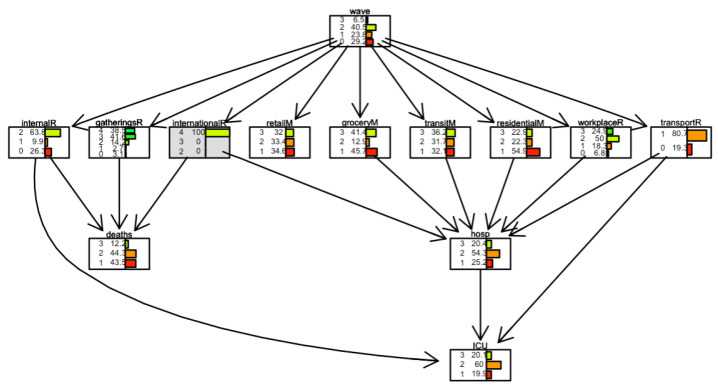
Scenario for Israel when international movements restriction is set to 4. The international movement restriction node is highlighted in grey.

Figure 20 and Figure 21 show two scenarios for Israel. As for Italy, in the first scenario, we set evidence as the lowest level of the *international movement restriction* variable. In the second scenario, we set evidence as the highest level of the *international movement restriction* variable.

In Israel, the impact of the *international movement restriction* variable is greater than in Italy; when restrictions are present, the number of days with a low number of daily deaths (less than 5) is 40%, compared to 12% when restrictions are not present.

**Figure 22:**
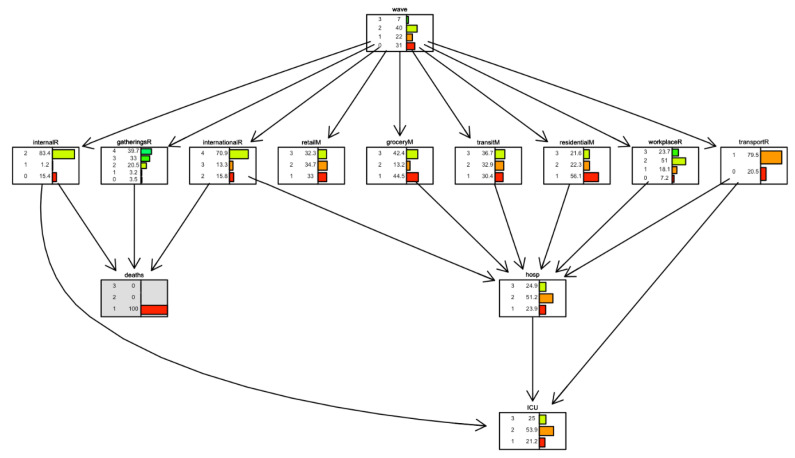
Scenario for Israel when daily deaths are less than 5 (level 1). Deaths node is highlighted in grey.

**Figure 23:**
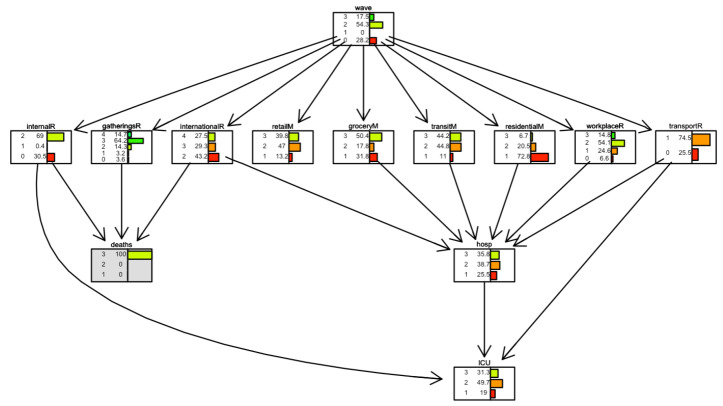
Scenario for Israel when daily deaths are more than 20 (level 3). The deaths node is highlighted in grey.

In Figure 22 and Figure 23, for Israel, inverse inference is applied, the daily deaths level remains fixed, and the model observes what happens in parent nodes. In Figure 22 the scenario is one where the number of daily deaths is always lower than 5; in Figure 23, the scenario is one where the number of daily deaths is always larger than 20. We observe, for example, that when international movement restriction was high (level 3), 70.9% of days, daily deaths were lower than 5. When daily deaths were more than 20, in only 27.5% of days international movement restriction was high (level 3).

## 6. Discussion

Given that COVID-19 is a human-to-human-transmitted disease, with high infection rates, pandemic mitigation policies should limit people’s physical interactions, but at the same time, allow for maximal continuity of economic activity. As a consequence, managing and applying movement restrictions should take into consideration three factors: (a) the type of the restriction (local, national, the activity being restricted); (b) the duration of the restriction; and (c) the severity of the morbidity. Gaining compliance and adherence to the restriction policies depends on these three factors. As the results in our analysis show, over time, the population compliance decreased in both countries, even when health indicators were not satisfying. This highlights the importance of applying a selective and differential approach in various public health conditions. For example, restricting international movement had a larger effect on the number of death cases. At the same time, other restrictions, such as ones applied to workplaces and transport stations, had a moderate effect on the number of hospitalised patients. Moreover, behavioural changes, such as attendance at grocery stores and pharmacies, workplace and transit stations, had a very small effect on health indicators. This means that people limit attendance at public places according to their personal risk assessment, while the consequences of such limitations vary according to the duration of the restrictions and their type. For example, we found that in Israel, greater numbers of death cases are followed by internal restrictions, i.e., lockdowns, but also higher attendance at public places, such as retail stores and transit stations. In Italy, higher death rates are the result of fewer restrictions, and higher levels of hospitalized patients.

The analysis also highlights the strong effect of internal and international travel restrictions on COVID-19 death rates. This is in comparison to other restrictions which have a smaller effect on the number of hospitalised patients. This finding is important as vaccinations and improved healthcare systems provide effective solutions that permit lower levels of restrictions and lower levels of population compliance with instructions and limitations. More importantly, it allows for a country to maintain its economic activity running with acceptable levels of morbidity and hospitalisations. However, it seems that international restrictions, such as airport closures, have a strong and direct effect on the number of deaths. In terms of mitigation policy, closing international borders appears to be an effective tool for a quick reduction of death cases, when the pandemic exceeds acceptable limits.

Although critical in the pandemic spread, population behaviour was found to have only a moderate direct effect on the health indicators. In fact, both countries have reacted in an opposite way at early stages of the pandemic. On February 2020, Italy was late in reacting, with no restrictions and extreme hospitalisations, ICU and death cases. In Israel, immediately after detecting the first cases, a national total lockdown was applied, which led to low levels of morbidity for few months. However, later, both countries experienced higher levels of morbidity with restrictions, but with lower levels of public compliance. This is important for policy decision-making considerations. Future pandemic management should assess the public’s ability and willingness to comply with directions. Apparently, the severity of the health indicators does not guarantee full compliance with the instructions. The effect of restrictions while the public does not comply is dramatic as reflected by higher levels of morbidity and death rates.

Dealing with a pandemic such as COVID-19 needs to account for possible multiple strategies. The continuous relationship over time among the variables involved renders an approach with BNs and SEM particularly suitable. The reversed conditional analysis described above is useful to “learn from experience”. Restriction measures can be successful or not. This is why we model a variable “wave” to temporally and spatially contextualise the restriction measures following, or preceding, the introduction of restriction measures and consequently affecting the behaviour of the population. The current analysis has several limitations. In general, we show the aggregated behaviour of variables with respect to key healthcare variables (hospitalisation, ICU and deaths).

Direct consequences of a measure put in place, at a certain time *t*, should be studied with a time series model. We construct scenarios regarding choices taken to overcome pandemic problems. These are summarized by counterfactual questions such as: “if in this situation one would have done this, what would have happened to this quantity?”. The COVID-19 pandemic is a continuous event that needs to be managed as such. The definition of a “wave”, in each country, reflects increased levels of health indicators during a certain period. Health data observatories and surveillance systems, across countries, need to calibrate the data and provide a multivariate perspective. In this paper, we provide examples of both by applying a methodology that can be generalised to individual locations.

## 7. Conclusions

In this article we presented a methodology to examine the mutual effect of national policy and population behaviour when a huge pandemic such as the COVID-19 one or, more in general, a global emergency, affects a country. This methodology was first applied on pandemic data from Italy and then on Israel data for a comparative and confirmatory analysis.

Starting from the multivariate structure in the daily COVID-19 indicators (new cases, number of hospitalisations, ICU admissions and COVID-19 deaths), the population activity and restriction data, we introduced a modelling approach based on complementary features involving an “ensemble-type” analysis combining outcomes from multiple models. We considered the impact of lockdown measures and mobility restrictions, as reflected by people’s behaviour and mobility trends, using a multimethod approach, combining Bayesian network analysis, structural equation modelling and ’what-if’ Bayesian network scenarios.

This approach might serve as an effective tool for policy makers evaluating anticipated restriction policies due to an emergency. Such assessments are dependent on health conditions, desired outcomes and variables such as population compliance over time and the national and local health system capacity.

## Figures and Tables

**Table 1:** Loadings, regressions and covariances for the Italian data (JMP Pro Version 16). * < 0.05.

Regressions	Estimate	SE	Prob>|Z|
Workplaces → [hospitalisation_lag10]	0.0051733	0.0013351	0.0001 *
Workplaces → [death_lag20]	−0.001852	0.0012394	0.1350
Workplace closing → [icu_lag15]	−0.030367	0.0278552	0.2756
Workplace closing → [hospitalizations _lag10]	0.0667816	0.0270191	0.0134 *
wave → workplaces	3.19686	0.5874759	<0.0001 *
wave → workplace closing	0.0754524	0.0217294	0.0005 *
wave → transportation closing	0.0837236	0.0126287	<0.0001 *
wave → transit stations	2.9688032	0.4265175	<0.0001 *
wave → [icu_lag15]	0.1807456	0.0274846	<0.0001 *
wave → [hospitalisation_lag10]	0.2486583	0.0245819	<0.0001 *
wave → [death_lag20]	0.4204896	0.1221507	0.0006 *
wave → school closing	−0.316598	0.0169194	<0.0001 *
wave → retail and recreation	3.7666174	0.59323	<0.0001 *
wave → residential	−1.129711	0.2063731	<0.0001 *
wave → international movement restrictions	0.0842087	0.0199013	<0.0001 *
wave → internal movement restrictions	0.0676107	0.0158063	<0.0001 *
wave → grocery and pharmacy	5.10812	0.5543045	<0.0001 *
wave → gathering restrictions	0.3068871	0.0409729	<0.0001 *
Transport closing → [icu_lag15]	0.1455176	0.0273859	<0.0001 *
Stringency index → [icu_lag15]	0.0067435	0.0038163	0.0772
Stay home restrictions → [hospitalisations _lag10]	0.0472529	0.022111	0.0326 *
[icu_lag15] → [death_lag20]	1.1659765	0.2939227	<0.0001 *
[hospitalisation _lag10] → [death_lag20]	−2.089182	0.410195	<0.0001 *
Retail and recreation → [hospitalisations _lag10]	−0.005147	0.0014207	0.0003 *
residential → [hospitalisations_lag10]	0.013011	0.004218	0.0020 *
parks → [hospitalisations_lag10]	0.0019993	0.0004296	<0.0001 *
International movement restrictions → [icu_lag15]	0.0630446	0.018092	0.0005 *
Internal movement restrictions → [icu_lag15]	0.1338373	0.0667171	0.0449 *
Internal movement restrictions → [hospitalisations _lag10]	0.230179	0.0574105	<0.0001 *
Internal movement restrictions → [death_lag20]	0.5626452	0.1869919	0.0026 *
Grocery and pharmacy → [hospitalisation _lag10]	0.000544	0.0008651	0.5294
Gatherings restrictions → [icu_lag15]	−0.063505	0.0407235	0.1189
Gatherings restrictions → [hospitalisations _lag10]	−0.061278	0.035522	0.0845
Gatherings restrictions → [death_lag20]	−0.141549	0.0740964	0.0561
**Covariances**	**Estimate**	**SE**	**Prob>|Z|**
behave ↔ health	−24.47497	8.5668423	0.0043 *
Grocery and pharmacy ↔ retail and recreation	87.867134	9.2979598	<0.0001 *
residential ↔ workplaces	−10.21344	1.4937324	<0.0001 *
Stay home restrictions ↔ internal movement restrictions	0.7703725	0.0643261	<0.0001 *
Stringency index ↔ internal movement restrictions	8.9560929	0.7747634	<0.0001 *
Stringency index ↔ stay-home restrictions	8.8102202	0.7537571	<0.0001 *
Stringency index ↔ transport closing	3.708656	0.3479073	<0.0001 *
workplaces ↔ parks	−275.3571	24.166104	<0.0001 *

**Table 2:** Summary of fit for the Italian data (JMP Pro version 16).

Sample Size	333
Iterations	77
−2 Log Likelihood	22,928.544
AIC	23,165.042
BIC	23,433.852
$\chi^{2}$	3148.5972
DF	102
Prob $>\chi^{2}$	0
CFI	0.7122846
TLI	0.5684269
NFI	0.7068871
Revised GFI	0.4951449
Revised AGFI	0.0645332
RMSEA	0.2994921
Lower 90%	0.2905408
Upper 90%	0.3085381
RMR	70.587903
SRMR	0.3716811

**Table 3:** Loadings, regressions and covariances for the Israeli data (JMP Pro Version 16). * < 0.05.

Regressions	Estimate	SE	Prob>|Z|
Gathering restrictions → [death_lag20]	0.0834036	0.0646139	0.1968
Grocery and pharmacy → [hospitalisations _lag10]	0.0073164	0.0025899	0.0047 *
Internal movement restrictions → [death_lag20]	0.2133128	0.0448712	<0.0001 *
Internal movement restrictions → [hospitalisation _lag10]	−0.070286	0.0681075	0.3021
Internal movement restrictions → [icu_lag15]	0.2298243	0.0530456	<0.0001 *
International movement restrictions → [death_lag20]	−0.494448	0.06866	<0.0001 *
International movement restrictions → [hospitalizations_lag10]	−0.550245	0.0422315	<0.0001 *
residential → [hospitalisation _lag10]	−0.029104	0.0083352	0.0005 *
Retail and recreation → [hospitalisation_lag10]	0.006643	0.0042186	0.1153
[hosp_lag10] → [death_lag20]	0.3147609	0.0827129	0.0001 *
[hosp_lag10] → Lag[icu_lag15]	0.4229745	0.0725468	<0.0001 *
Transit stations → [hospitalisation_lag10]	−0.041554	0.0071265	<0.0001 *
Transit stations → [icu_lag15]	−0.003296	0.002778	0.2354
Transport closing → [hospitalisation_lag10]	0.6199998	0.0924994	<0.0001 *
Transport closing → [icu_lag15]	−0.352726	0.0735336	<0.0001 *
wave → gathering restrictions	0.2335669	0.026049	<0.0001 *
wave → international movement restrictions	−0.142389	0.0416318	0.0006 *
wave → parks	1.7042613	1.3502963	0.2069
wave → [hospitalisation_lag10]	0.2830623	0.0341744	<0.0001 *
wave → transport closing	0.0786077	0.0205476	0.0001 *
wave → workplace closing	0.1612515	0.0261237	<0.0001 *
Workplace closing → [hospitalisation_lag10]	0.3268637	0.1029741	0.0015 *
Workplace closing → [icu_lag15]	0.0525362	0.0701275	0.4538
**Covariances **	**Estimate**	**SE**	**Prob>|Z|**
behavior ↔ health	1.8928684	0.4624537	<0.0001 *
Gatherings restrictions ↔ behaviour	−15.63618	1.381421	<0.0001 *
Internal movement restrictions ↔ workplace closing	0.5582769	0.0497297	<0.0001 *
residential ↔ grocery and pharmacy	21.020041	2.8550311	<0.0001 *
[icu_lag15] ↔ international movement restrictions	−0.285663	0.0450603	<0.0001 *
Transit stations ↔ transport closing	0.7953274	0.1034623	<0.0001 *
wave ↔ transit stations	−2.446618	0.3286085	<0.0001 *

**Table 4:** Summary of fit for the Israeli data (JMP Pro version 16).

Sample Size	333
Iterations	77
−2 Log Likelihood	17853.737
AIC	18,018.986
BIC	18,237.075
$\chi^{2}$	1035.5668
DF	53
Prob $>\chi^{2}$	$2.93\times 10^{-182}$
CFI	0.8006927
TLI	0.6577931
NFI	0.7937491
Revised GFI	0.7028438
Revised AGFI	0.3328002
RMSEA	0.2359506
Lower 90%	0.2235195
Upper 90%	0.2486080
RMR	8.5534396
SRMR	0.1926672

## Data Availability

The dataset used in this research are available on request.
